# Decreased Time to Treatment Initiation for Multidrug-Resistant Tuberculosis Patients after Use of Xpert MTB/RIF Test, Latvia 

**DOI:** 10.3201/eid2203.151227

**Published:** 2016-03

**Authors:** Helen R. Stagg, Peter J. White, Vija Riekstiņa, Andra Cīrule, Ģirts Šķenders, Vaira Leimane, Liga Kuksa, Gunta Dravniece, James Brown, Charlotte Jackson

**Affiliations:** University College London, London, UK (H.R. Stagg, J. Brown, C. Jackson);; Medical Research Council Centre for Outbreak Analysis and Modelling, London (P.J. White);; National Institute for Health Research Health Protection Research Unit in Modelling Methodology, London (P.J. White);; Imperial College School of Public Health, London (P.J. White);; Public Health England, London (P.J. White);; University of Latvia, Riga, Latvia (V. Riekstiņa);; Riga East University Hospital, Riga (V. Riekstiņa, A. Cīrule, Ģ. Šķenders, V. Leimane, L. Kuksa);; KNCV Tuberculosis Foundation, The Hague, the Netherlands (G. Dravniece);; Royal Free London National Health Service Trust, London (J. Brown)

**Keywords:** tuberculosis and other mycobacteria, bacteria, antimicrobial resistance, multidrug resistance, molecular diagnostics, rifampin, time to treatment initiation, pulmonary, multidrug-resistant tuberculosis, MDR TB, Xpert MTB/RIF, Latvia

## Abstract

Few studies have examined whether the Xpert MTB/RIF test improves time to treatment initiation for persons with multidrug-resistant tuberculosis (MDR TB). We determined the impact of this test in Latvia, where it was introduced in 2010. After descriptive analyses of pulmonary MDR TB patients in Latvia during 2009–2012, time to treatment initiation was calculated, and univariate and multivariable accelerated failure time models were constructed. Univariate results showed strong evidence of an association between having rifampin-resistant TB detected by Xpert MTB/RIF and reduced time to treatment initiation versus the test not being used. A multivariable model stratifying by previous TB showed similar results. Our finding that in Latvia, time to treatment initiation was decreased for MDR TB cases that were rifampin-resistant TB by XpertMTB/RIF has implications for the use of this test in other settings with a high burden of MDR TB in which rifampin resistance is highly predictive of MDR TB.

## Abstract 2

This test decreased time to treatment initiation by 66%–84%.

Timely diagnosis and treatment of multidrug-resistant tuberculosis (MDR TB), of which there were an estimated 480,000 cases in 2014, have been identified as a critical challenge for TB control (*1**,**2*). In 2010, the World Health Organization endorsed the Xpert MTB/RIF (*Mycobacterium tuberculosis*/rifampin test, hereafter referred to as Xpert; Cepheid, Sunnyvale, CA, USA) as a rapid test for the diagnosis of TB, including rifampin-resistant cases, citing a strong recommendation in their 2013 policy update for its use “as an initial diagnostic test in individuals suspected of having MDR TB or HIV-associated TB” (*3**,**4*).

Implications of the implementation of Xpert for a country are closely linked to where in the clinical pathway it is placed and how clinicians view the technology. Although many studies (and reviews) have assessed sensitivity, specificity, and predictive values of Xpert (*5*), few studies have examined the impact of this technology on the rapidity with which appropriate treatment is given to TB patients (*6**–**11*). Even fewer studies have assessed the effects of Xpert on time to MDR TB treatment initiation for MDR TB patients. In South Africa, a reduction in time from first diagnostic sputum collection to treatment commencement from 43 days to 17 days was found when an algorithm based on Xpert was compared with an algorithm based on the line probe assay (LPA) (*12*).

Despite successes in TB and MDR TB control in recent years, Latvia is classified as having a high burden of MDR TB; in 2014, 8.2% of new TB cases and 30% of re-treatment TB cases were estimated to be MDR (*2**,**13*). Absolute case numbers (48 new and 37 re-treatment in 2014) are relatively low, which is a reflection of TB incidence and population size (*13*). A total of 99% of new cases and 86% of re-treatment cases were reported by the World Health Organization to have been tested phenotypically or genotypically for rifampin-resistant or MDR TB in 2014; the 86% reflects clinical use of prior drug-susceptibility results for re-treatment cases. All MDR TB cases were tested for resistance to second-line drugs in 2013 (*14*). Access to MDR TB treatment is universal in Latvia. Most MDR TB patients are hospitalized, at least for the initial treatment period, in Riga, the capital of Latvia.

Latvia has 2 Xpert systems, both in Riga, where Xpert has been used since 2010. Xpert was initially targeted toward groups at high risk for MDR TB (e.g., contacts of persons with MDR TB). Since 2012, wider use of Xpert was promoted (e.g., for re-treatment cases). Two diagnostic pathways for MDR TB patients were used (Figure 1). When Xpert was available, its results determined whether a patient was initially given treatment for MDR TB. In the absence of Xpert, patients were initially given first-line drugs. In Latvia, rifampin resistance is a good predictor of MDR TB (*15*). The positive predictive value (PPV) of rifampin resistance for MDR is partly determined by MDR TB prevalence (at an MDR TB prevalence >14.2% for new TB cases and >20% for re-treatment cases, the PPV is 90%) (*16*).

**Figure 1:**
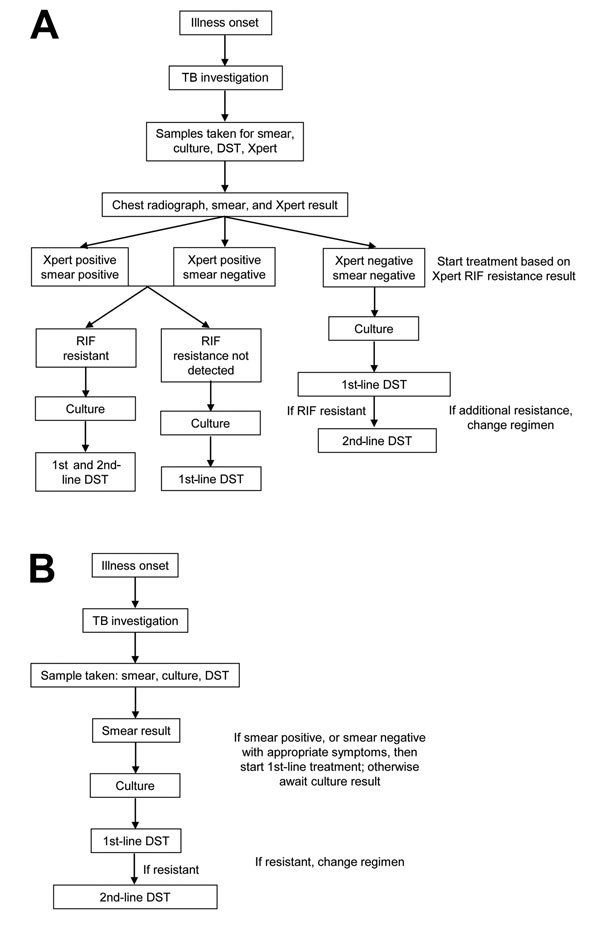
Diagnostic pathways for patients with multidrug-resistant tuberculosis, Latvia, 2012. A) With use of Xpert MTB/RIF; B) without use of Xpert MTB/RIF. A line probe assay was used if Xpert and DST showed discordant results. MTB, *Mycobacterium tuberculosis*; RIF, rifampin; TB, tuberculosis; DST, drug sensitivity testing; Xpert, Xpert MTB/RIF.

We used surveillance data to examine the relationship between use of Xpert and time to treatment initiation for MDR TB patients in Latvia. We aimed to provide useful data for clinicians and policy makers evaluating implementation of Xpert in settings with a high burden of MDR TB in which rifampin resistance is a good indicator of MDR TB.

## Methods

The treatment cohorts for MDR TB patients in Latvia during 2009–2012, excluding cases in prisoners, were obtained from the national MDR TB surveillance system, together with their demographic and clinical data (supplemented from paper records where necessary). Persons with only extrapulmonary MDR TB were excluded, which is consistent with primary use of Xpert. Ethical approval for the study was provided by the ethics committee of Riga Stradins University. Informed consent was not required because this study used an anonymous surveillance dataset.

Age was grouped into 20-year categories (<20, 20–39, 40–59, and >60 years); nationality as Latvian or other; residency region in Latvia as Riga or elsewhere (consistent with diagnostic methods available in Riga); and social risk factors (history of imprisonment, history of or current drug abuse, current homelessness, current dependence on alcohol) into a single dichotomous variable of >1 risks versus none or unknown. Site of disease was classified as pulmonary or pulmonary and extrapulmonary and HIV status as positive, negative, or unknown. Use of Xpert was categorized as not conducted versus conducted for descriptive analysis; the conducted category was subdivided into conducted and rifampin-resistant TB and conducted and negative result for rifampin-resistant TB. History of TB and sex were treated as binary variables. Reporting date was grouped into year of reporting.

Time between date patient samples were obtained and date of MDR TB treatment initiation (start of MDR TB treatment was defined as start of use of second-line drugs) was calculated and used as the scale (time since entry) for regression analysis. Persons for whom either date was missing were excluded. When persons started MDR TB treatment on the day that their samples were obtained, time to treatment initiation was set to 0.25 days for regression analysis.

**Figure 2:**
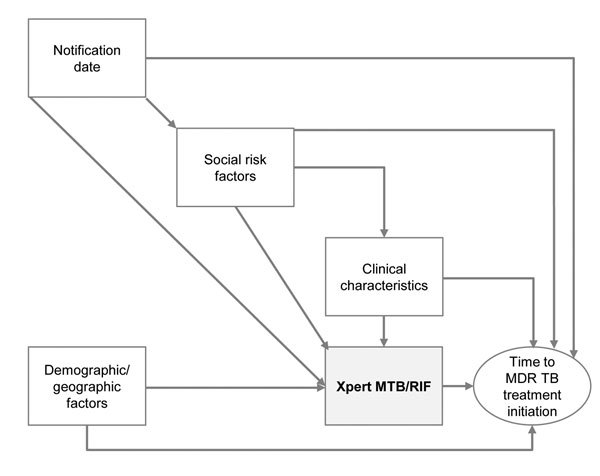
Conceptual framework of relationship between use of Xpert MTB/RIF and time to treatment initiation among patients with multidrug-resistant tuberculosis (MDR TB), Latvia, 2009–2012. Demographic and geographic variables were sex, age, country of birth, and region of Latvia. Clinical variables were previously having had tuberculosis, site of disease, and HIV status. Social risk factor variables were history of imprisonment, history of or current drug abuse, current homelessness, current dependence on alcohol. MTB, *Mycobacterium tuberculosis*; RIF, rifampin.

Demographic and clinical characteristics of patients were described, and time-to-event (MDR TB treatment initiation) data plotted, including using Kaplan-Meier plots to examine the proportion of persons who had completed treatment at given time points. Before regression modeling, we created a conceptual framework of the relationship between main exposure (Xpert use) and outcome (time to initiation of MDR TB treatment) (Figure 2) and used this framework to identify a priori and potential confounders, as well as effect modifiers (*17*).

We initially assessed use of linear regression to measure the effect of Xpert use on time to treatment initiation. However, the highly skewed distribution of time to treatment initiation (even after log or reciprocal transformation) showed that this technique was not appropriate. Accelerated failure time (AFT) models were chosen as an alternative technique. These models assume that the fraction of persons surviving (i.e., not receiving appropriate treatment) in 1 group at any given time point is proportional to the equivalent fraction of the other group.

We tested this assumption by using quantile–quantile plots, which plot quantiles of survival distribution for the exposed group against those for the unexposed group. AFT models are considered appropriate if these plots approximate a straight line. We then used a univariate model to calculate the time ratio, 95% CI, and p value for the association between Xpert use and time to MDR TB treatment initiation. The time ratio represents the relative time to MDR TB treatment initiation between groups (i.e., a ratio of 0.9 indicates that persons in the exposed [tested] group are 10% more likely to have initiated treatment than those in the unexposed [not tested] group). In a scenario in which everyone initiates treatment, a ratio of 0.9 indicates a 10% reduction in time to initiation among exposed persons. An exponential distribution of survival times was assumed; a Weibull distribution was also investigated in preliminary analyses, but was found to fit the data poorly.

Exposures classified as a priori confounders (sex, age, previous TB) and potential confounders associated with outcome and Xpert use, but not on the causal pathway between these 2 factors, were included in the initial multivariable model. We used a backward deletion strategy in which potential confounders were individually removed and the full (all potential confounders present) and reduced (1 potential confounder removed) models were compared by using square roots of estimated mean squared errors (*18**,**19*). Thus, a final model was derived while simultaneously enabling assessment of confounding, multicollinearity and maintenance of a priori confounders.

Likelihood ratio tests were conducted to test for linearity (age, year) and effect modification. Region and previous TB were pre-identified as potential effect modifiers. Year of reporting was excluded from initial modeling so that data for 2009 could be used, but was planned to be included as a confounder during sensitivity analysis. Analysis was conducted by using Stata 13 (StataCorp LP, College Station, TX, USA) and Excel (Microsoft, Redmond, WA, USA) software.

## Results

Of 398 persons in treatment cohorts in Latvia during 2009–2012, five persons were excluded because of missing dates and 6 more were excluded because they had only extrapulmonary disease. This exclusion resulted in 387 pulmonary TB patients, of whom 262 did not have Xpert testing (100% for 2009, 84% for 2010, 45% for 2011, and 39% for 2012), 110 had rifampin-resistant TB by Xpert, and 15 had negative results for rifampin-resistant TB by Xpert (11 in which TB was not detected, 3 in which rifampin resistance was not detected, and 1 in which rifampin sensitivity was not determined). These 15 patients were subsequently found to have MDR TB culture and after phenotypic testing. Of 387 patients, 295 (76%) were male, 355 (92%) were Latvian, 239 (62%) lived outside Riga, 294 (76%) were HIV negative, and 348 (90%) had only pulmonary TB (Table 1). Data for current homelessness was missing for 1 person. Factors associated with use of Xpert appeared to be notification year, region, having previously had TB, site of disease, and HIV status (Table 1).

**Table 1:** Descriptive analysis of 387 patients in MDR TB treatment cohorts, Latvia, 2009–2012*

Variable	Total, no. (%)	Xpert MTB/RIF, no. (%)		
		Not conducted	Conducted, rifampin-resistant TB	Conducted, negative result
Overall exposure	387 (100.0)	262 (67.7)	110 (28.4)	15 (3.9)
Xpert MTB/RIF				
Not conducted	262 (67.7)	NA	NA	NA
Conducted, rifampin-resistant TB	110 (28.4)	NA	NA	NA
Conducted, negative result	15 (3.9)	NA	NA	NA
Year reported				
2009	114 (29.5)	114 (100.0)	0	0
2010	80 (20.7)	67 (83.8)	10 (12.5)	3 (3.8)
2011	91 (23.5)	41 (45.1)	45 (49.5)	5 (5.5)
2012	102 (26.4)	40 (39.2)	55 (53.9)	7 (6.9)
Sex				
M	295 (76.2)	200 (67.8)	88 (29.8)	7 (2.4)
F	92 (23.8)	62 (67.4)	22 (23.9)	8 (8.7)
Age, y				
<20	5 (1.3)	4 (80.0)	1 (20.0)	0
20–39	151 (39.0)	92 (60.9)	53 (35.1)	6 (4.0)
40–59	190 (49.1)	135 (71.1)	47 (24.7)	8 (4.2)
≥60	41 (10.6)	31(75.6)	9 (22.0)	1 (2.4)
Country of birth				
Latvia	355 (91.7)	241 (67.9)	100 (28.2)	14 (3.9)
Other	32 (8.3)	21 (65.6)	10 (31.3)	1 (3.1)
Region of Latvia				
Riga	148 (38.2)	83 (56.1)	56 (37.8)	9 (6.1)
Other	239 (61.8)	179 (74.9)	54 (22.6)	6 (2.5)
Social risk factors				
None or unknown	201 (51.9)	137 (68.2)	58 (28.9)	6 (3.0)
>1	186 (48.1)	125 (67.2)	52 (28.0)	9 (4.8)
Previous TB				
No	255 (65.9)	154 (60.4)	90 (35.3)	11 (4.3)
Yes	132 (34.1)	108 (81.8)	20 (15.2)	4 (3.0)
Site of disease				
Pulmonary	348 (89.9)	245 (70.4)	91 (26.1)	12 (3.4)
Pulmonary and extrapulmonary	39 (10.1)	17 (43.6)	19 (48.7)	3 (7.7)
HIV status				
Negative	294 (76.0)	210 (71.4)	74 (25.2)	10 (3.4)
Positive	56 (14.5)	24 (42.9)	29 (51.8)	3 (5.4)
Unknown	37 (9.6)	28 (75.7)	7 (18.9)	2 (5.4)

*Prisoners with MDR TB and persons with extrapulmonary MDR TB were excluded. MDR TB, multidrug-resistant tuberculosis; MTB, *Mycobacterium tuberculosis*; RIF, rifampin; NA, not applicable.

**Figure 3:**
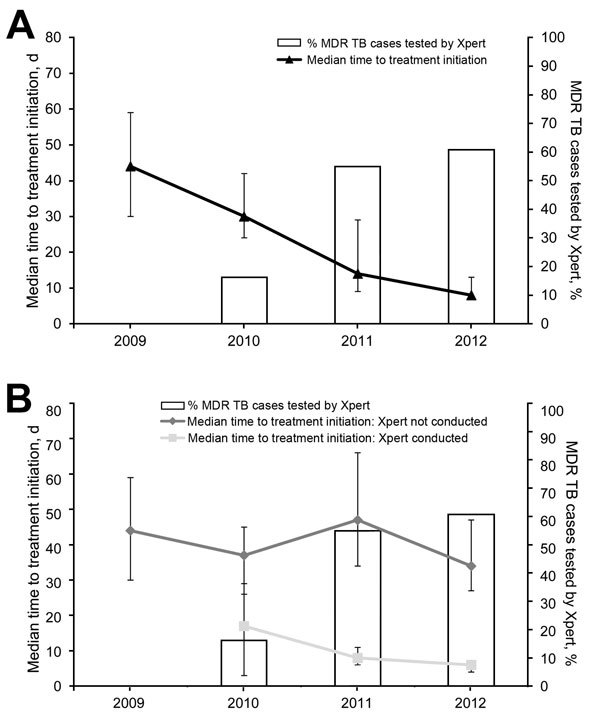
Relationship between use of Xpert MTB/RIF (Xpert) and time to treatment initiation among patients with multidrug-resistant tuberculosis (MDR TB) Latvia, 2009–2012. Shown are percentages of MDR-TB patients that underwent Xpert MTB/RIF testing (bars) and median time to treatment initiation (lines) with binomial distribution–derived CIs (error bars) for A) all patients and B) patients with and without testing by Xpert. MTB, *Mycobacterium tuberculosis*; RIF, rifampin.

For the overall cohort, median time from the date samples were obtained to MDR TB treatment initiation was 27 (95% CI 22–30, range 0–385) days. Sixteen (4%) patients started treatment on the day samples were obtained. When Xpert was not used, median delay was 40 (95% CI 33–45) days. When Xpert was used, median delay was 7 (95% CI 6–8) days. Among persons for whom Xpert was used, median delay was 6 (95% CI 5–7) days when Xpert detected persons to have rifampin-resistant TB and 57 (95% CI 28–99) days when Xpert showed negative results. Overall, median time to treatment initiation in Latvia decreased over time (Figure 3, panel A) and was relatively consistent for persons for whom Xpert was not used, which indicated that additional persons selected for Xpert testing in 2012 were not different in terms of their risk for a longer time to MDR TB treatment initiation than the initial population selected (Figure 3, panel B). These unadjusted figures suggest that time to MDR TB treatment initiation was shorter in persons who underwent Xpert testing (Figure 3, panel B).

**Figure 4:**
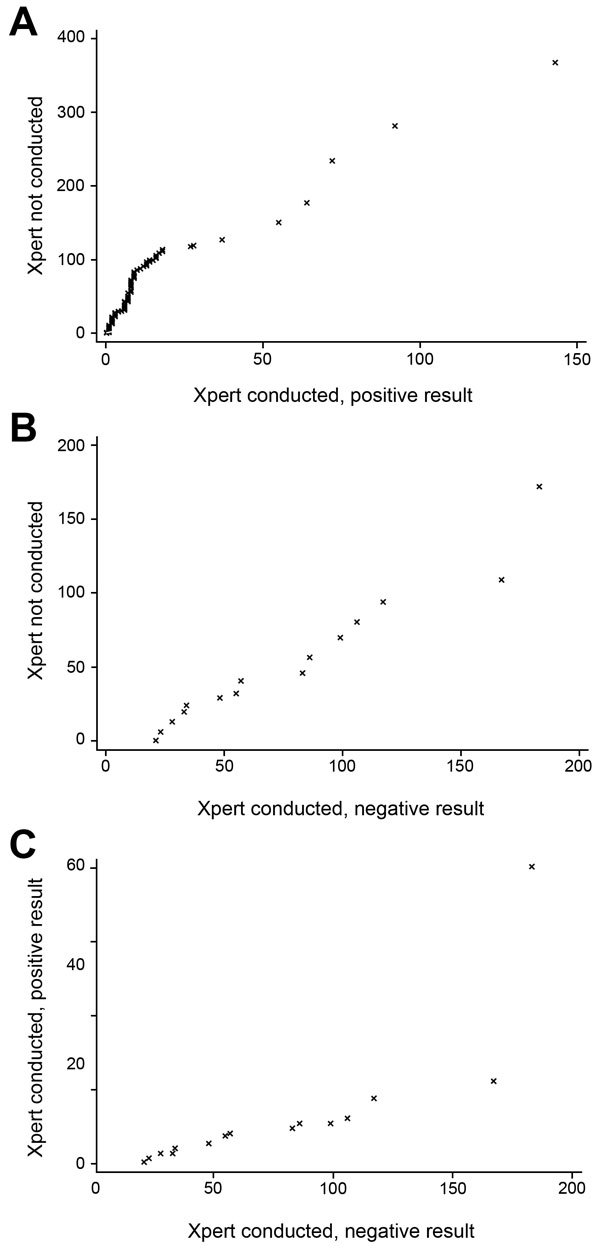
Quantile–quantile plots of time to multidrug-resistant tuberculosis (MDR TB) treatment initiation by use and results of Xpert MTB/RIF (Xpert) for patients with MDR TB, Latvia, 2009–2012. Shown are time to MDR TB treatment initiation (days) for patients A) who were not tested by Xpert vs. those who had rifampin-resistant TB by Xpert, B) those who were not tested vs. those who had a negative result for rifampin-resistant TB, and C) those who were tested by Xpert and had positive vs. negative results for rifampin-resistant TB. MTB, *Mycobacterium tuberculosis*; RIF, rifampin.

A quantile−quantile plot comparing persons for whom Xpert was not used with those who had rifampin-resistant TB by Xpert showed a linear relationship, which supported use of the AFT model (Figure 4, panel A). Linearity could only be tenuously assessed when persons who had negative results by Xpert were compared with either of the other groups. The final data quantile in both instances was highly influential and outlying in each plot, but could not be ignored because of the small number of data points (Figure 4, panels B, C). Given the small number of persons in this third group, their unexpected test results, and that they were likely to have had a different mechanism for a change in the time frame to initiating treatment, they were excluded from the main analysis, which resulted in 372 patients.

**Figure 5:**
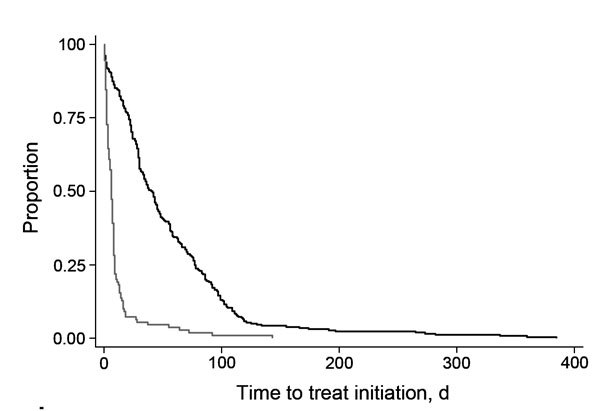
Kaplan-Meier plot of time to treatment initiation by use and results of Xpert MTB/RIF in patients with multidrug-resistant tuberculosis (MDR TB), Latvia, 2009–2012. Shown is time to MDR TB treatment initiation (days) for patients who were not tested by Xpert MTB/RIF (dark gray line) and those who had rifampin-resistant TB by Xpert MTB/RIF (light gray line). MTB, *Mycobacterium tuberculosis*; RIF, rifampin.

A Kaplan-Meier plot of time to MDR TB treatment initiation (Figure 5) indicated a pattern similar to that shown in Figure 3, panel B. This plot shows that persons with rifampin-resistant TB by Xpert began treatment sooner than those who did not have Xpert testing.

For the 372 patients, overall median time to diagnosis was 24 (95% CI 21–29) days (Table 2). A univariate AFT model showed strong evidence for an association between having rifampin-resistant TB by Xpert (baseline: Xpert not done) and reduced time to MDR TB treatment initiation (time ratio 0.19, 95% CI 0.15–0.23, p<0.001; baseline median in model 38 days) (Table 2). This finding corresponded to an absolute reduction of ≈31 days. HIV status also showed strong evidence for an association with time to treatment initiation. There was strong evidence that persons having extrapulmonary and pulmonary TB, having >1 social risk factor, and living in Riga led to quicker treatment initiation than for having only pulmonary TB, being without such risk factors, and living outside Riga, respectively.

**Table 2:** Univariate and multivariable accelerated failure time models of association between Xpert MTB/RIF use and time to MDR TB treatment initiation for patients in MDR TB treatment cohorts, Latvia, 2009–2012*

Variable	Crude analysis, median days to diagnosis (95% CI)	Univariate regression			Multivariable regression	
		Median days to diagnosis	p value, time ratio (95% CI)		Median days to diagnosis	p value, time ratio (95% CI)
Overall	24 (21–29)	NA	NA		NA	NA
Xpert MTB/RIF						
Not conducted	40 (33–45)	38	<0.001		†	†
Conducted, rifampin-resistant TB	6 (5–7)	NA	0.19 (0.15–0.23)		†	†
Sex						
M	26 (21–30)	30	0.08		30	0.08
F	21 (11–30)	NA	0.80 (0.63–1.02)		NA	0.79 (0.62–1.02)
Age, y						
<20	64 (2−‡)	29	0.98		33	0.78
20–39	21 (9–29)	NA	1.00 (0.87–1.16)		NA	0.98 (0.84–1.14)
40–59	28 (22–30)	NA	NA		NA	NA
>60	30 (14–48)	NA	NA		NA	NA
Country of birth						
Latvia	26 (21–30)	29	0.63		NA	NA
Other	14 (8–29)	NA	0.91 (0.63–1.32)		NA	NA
Region of Latvia						
Riga	16 (11–24)	NA	0.78 (0.63–0.96)		NA	0.93 (0.75–1.16)
Other	30 (24–36)	32	0.02		32	0.51
Social risk factors						
None or unknown	30 (23–35)	32	0.03		NA	NA
>1	22 (11–28)	NA	0.79 (0.65–0.97)		NA	NA
Previous TB						
No	24 (18–29)	28	0.39		†	†
Yes	27 (16–35)	NA	1.10 (0.89–1.36)		†	†
Site of disease						
Pulmonary	28 (23–31)	30	0.01		30	0.05
Pulmonary and extrapulmonary	7 (4–11)	NA	0.61 (0.43–0.86)		NA	0.69 (0.49–0.98)
HIV status						
Negative	29 (24–33)	31	0.003		NA	NA
Positive	7 (4–11)	NA	0.59 (0.44–0.79)		NA	NA
Unknown	29 (14–48)	NA	1.00 (0.71–1.43)		NA	NA

*Crude median time to MDR TB treatment initiation was determined (with a binomially derived CI), followed by association between use of Xpert MTB/RIF and time to treatment initiation in univariate and multivariable (372 patients) accelerated failure time models and median time to treatment in such models. Prisoners with MDR TB and persons with only extrapulmonary MDR TB were excluded; 15 patients with negative results by Xpert MTB/RIF were also excluded. Age was treated as a linear variable in regression analyses. The final regression model adjusted for all variables except country of birth, social risk factors, and HIV status. MDR TB, multidrug-resistant tuberculosis; MTB, *Mycobacterium tuberculosis*; RIF, rifampin; NA, not applicable. †Stratified results are shown in Table 3. ‡No binomial prediction as small stratum.

Region, site of disease, and HIV status were associated with the outcome and main exposure and were included with a priori confounders (sex, age, and previous TB) in an initial multivariable model. The backwards deletion strategy removed HIV status. Age was included as a linear variable (p = 0.42, by test for linearity). Region was not an effect modifier (p = 0.44, by test for interaction), unlike having previously had TB (p = 0.01, by test for interaction). Effect estimates for the effect of Xpert are therefore presented stratified by previous TB status (Table 3). Having extrapulmonary and pulmonary TB, and being a female patient were weakly associated with the outcome in the multivariable model, with a reduction in the time to treatment initiation (Table 2).

**Table 3:** Stratified results for multivariable accelerated failure time model of association between Xpert MTB/RIF use and time to MDR TB treatment initiation for patients in MDR TB treatment cohorts, Latvia, 2009–2012*

Exposure	Stratifier	Multivariable regression, time ratio (95% CI)	p value
Xpert MTB/RIF	Previous TB		
Not conducted	No	Baseline	
Conducted, rifampin-resistant TB	No	0.16 (0.12−0.21)	<0.001
Not conducted	Yes	Baseline	
Conducted, rifampin-resistant TB	Yes	0.34 (0.21–0.55)	<0.001
Previous TB	Xpert MTB/RIF		
No	Not conducted	Baseline	
Yes	Not conducted	0.80 (0.62–1.04)	0.09
No	Conducted, rifampin-resistant TB	Baseline	
Yes	Conducted, rifampin-resistant TB	1.69 (1.03–2.76)	0.04

*Association between use of Xpert MTB/RIF and time to MDR TB treatment initiation was stratified by the effect modifier previous tuberculosis. Association between previous tuberculosis and time to MDR TB treatment initiation was stratified by Xpert use. There were 372 patients in the model. All results derived from the final model shown in Table 2. Median in baseline strata of Xpert MTB/RIF and previous tuberculosis was 41 days. MTB, *Mycobacterium tuberculosis*; RIF, rifampin. MDR TB, multidrug-resistant tuberculosis; NA, not applicable.

The effect of Xpert on time to MDR TB treatment initiation for persons who had not previously had TB differed little from the effect found by univariate analysis in the fully adjusted model (time ratio 0.16, 95% CI 0.12–0.21, Wald p<0.001; median 41 days in baseline strata of Xpert) (Table 3). A smaller time reduction was seen for persons who had previously had TB (time ratio 0.34, 95% CI 0.21–0.55, Wald p<0.001; median 34 days in baseline strata of Xpert). Thus, in this fully adjusted and stratified model, time to treatment initiation among persons found to have rifampin-resistant TB by Xpert was reduced by 35 days among patients who had previously had TB and by 22 days among those who had not previously had TB.

We found weak evidence for an association between previously having had TB and time to MDR TB treatment initiation in persons who had not had Xpert testing (time ratio 0.80, 95% CI 0.62–1.04, Wald p = 0.09). Evidence was stronger among persons found to have rifampin-resistant TB by Xpert (effect estimate 1.69, 95% CI 1.03–2.76, Wald p = 0.04).

A sensitivity analysis was conducted to determine the effect of including year as an additional confounder in the model (Figure 2), although time to treatment initiation in the absence of Xpert testing did not appear to have changed during 2010–2012 (Figure 3, panel B). This inclusion reduced overall power because Xpert was not used in 2009 (258 patients). Including year (as a categorical variable; p for linearity <0.001) had little impact on the effect estimate for the effect of Xpert (no previous TB 0.15, 95% CI 0.11–0.21, Wald p<0.001; previous TB 0.45, 95% CI 0.27–0.77, Wald p = 0.003; baseline median 41 days) (Table 4). Effect estimates per year were 1.43 (95% CI 1.00–2.03) for 2011 and 0.74 (95% CI 0.53–1.03) for 2012 (overall p<0.001; baseline 2010).

**Table 4:** Sensitivity analyses for findings of multivariable accelerated failure time model for association between Xpert MTB/RIF use and time to MDR TB treatment initiation for patients in MDR TB treatment cohorts, Latvia, 2009–2012*

Exposure: Xpert MTB/RIF	Stratifier: previous TB	Time ratio (95% CI), p value		
Model 1: 2-Strata Xpert, year as confounder	Model 2: 3-Strata Xpert	Model 3: 3-Strata Xpert, year as confounder
Not conducted	No	Baseline	Baseline	Baseline
Conducted, rifampin-resistant TB	No	0.15 (0.11−0.21), <0.001	0.16 (0.12−0.21), <0.001	0.15 (0.11−0.20), <0.001
Conducted, negative result	No	NA	1.17 (0.62−2.22), 0.62	1.09 (0.57−2.09), 0.80
Not conducted	Yes	Baseline	Baseline	Baseline
Conducted, rifampin-resistant TB	Yes	0.45 (0.27−0.77), 0.003	0.33 (0.20−0.54), <0.001	0.45 (0.27−0.76), 0.003
Conducted, negative result	Yes	NA	2.97 (1.07−8.28), 0.04	3.63 (1.27−10.37), 0.02

*Three models of sensitivity of the association between Xpert MTB/RIF use and the time to MDR TB treatment initiation stratified by the effect modifier previous TB were used. Model 1 included year as a confounder (restricting the dataset to 2010–2012; 258 patients). Model 2 included persons who had negative results for rifampin resistance by Xpert MTB/RIF (387 patients). Model 3: models 1 and 2 assessed simultaneously (273 patients). A final model was adjusted for all variables except country of birth, social risk factors, and HIV status (plus year where stated). Median in baseline strata of Xpert MTB/RIF and previous tuberculosis was 41 days for all analyses. A positive result was rifampin-resistant TB, and a negative result was rifampin sensitivity or no TB detected. MTB, *Mycobacterium tuberculosis*; RIF, rifampin. MDR TB, multidrug-resistant tuberculosis; NA, not applicable.

Inclusion of the 15 patients with MDR TB identified by phenotypic drug sensitivity testing, but not identified as having rifampin-resistant TB by Xpert, enabled Xpert results to be modeled as a 3-tiered variable. This inclusion had little influence on the effect estimate compared with analysis of Xpert use in 2 strata (Xpert not used vs. Xpert used and a positive result for rifampin-resistant TB) (Table 4). Having a negative result by Xpert was weakly associated with longer time to treatment initiation in persons who had previously had TB (effect estimate 2.97, 95% CI 1.07–8.28, Wald p = 0.04; baseline median 41 days), but this association was not seen for persons who had not previously had TB. Running such a model with year included as a confounder (273 patients) yielded similar results, and the previous association appeared stronger (effect estimate 3.63, 95% CI 1.27–10.37, Wald p = 0.02; baseline median 41 days) (Table 4).

## Discussion

An unadjusted AFT model showed strong evidence for an association between having rifampin-resistant TB detected by Xpert and a reduction of ≈1 month in time to treatment initiation (time ratio 0.19, 95% CI 0.15–0.23, p<0.001; baseline median in model 38 days) for patients with MDR TB in Latvia during 2009–2012. In a fully adjusted model, this relationship was supported, although the effect of Xpert was smaller for persons who had previously had TB than for those with had not had TB. This finding corresponded to a reduction in median time to treatment initiation of 35 days for persons who had not previously had TB and 22 days for those who had TB. This reduction is paralleled by a potential lengthening of the timeframe in persons with negative Xpert results who had previously had TB, although our estimates for these persons are highly uncertain. Inclusion of year as a confounder had little effect on the results obtained, which justifies our use of data for 2009 (when Xpert was not available in Latvia).

Data completeness in this cohort was high; thus, bias caused by missing data was not a major concern. Poor recording of dates causing measurement error, where present, was more likely to have been non-differential than differential, reducing precision around the effect estimate. Five patients had no recorded sampling date, but they represented a small fraction of the cohort. Patients with pulmonary TB for whom Xpert was used were likely to have been those producing sputum (i.e., potentially quicker to give a diagnosis of drug-resistant TB even without Xpert testing), although absence of an increase in time to diagnosis among persons for whom Xpert was not used seemed to negate this likelihood. We examined the impact of time to initiation of any MDR TB treatment regimen rather than a regimen tailored to second-line drug sensitivity testing. Given known high levels of additional drug resistance in MDR TB patients in Latvia, it is probable that many patients included in this study had their treatment regimen subsequently altered, which is not captured here and could be the subject of future research (*14*).

In South Africa, Naidoo et al. compared use of Xpert-based and LPA-based algorithms and found a decrease of 25 days in time to MDR TB treatment commencement when Xpert-based algorithms were used (*12*). Although the absolute size of such a decrease is highly context specific to the diagnostic pathway and available resources in each setting, this finding is consistent with our results.

The smaller improvement in time to treatment in persons who had previously had TB and who showed rifampin resistance by Xpert is assumed to be because these persons would be more likely to receive expedited MDR treatment even without use of Xpert. Few patients with MDR TB were not given a diagnosis of rifampin-resistant TB by Xpert (their mechanism for a differential timeframe to start treatment for MDR TB is probably different than that for other patient categories regardless, which made interpretations across strata of Xpert more difficult). However, the suggested increased time to MDR TB treatment initiation in this group when persons had previously had TB appears to indicate that clinicians in Latvia trust Xpert results to the extent that clinical suspicion of MDR TB, which might expedite starting treatment for MDR TB in the absence of Xpert results, is overridden. Such delays could have negative consequences for patients in terms of having a successful treatment outcome and highlights the need for accurate clinical judgement in diagnostic algorithms.

Latvia also has a few TB cases each year that have discordant results in the opposite direction. A total of 7 patients in 2013 had rifampin-resistant TB by Xpert, but 5 (3 of which showed rifampin resistance by LPA) had rifampin-sensitive results by BACTEC (Becton, Dickinson and Company, Franklin Lakes, NJ, USA), and 2 were not culture positive by BACTEC or on solid media.

Sensitivity of Xpert for detection of rifampin resistance has been documented in many settings, and specific mutations and mixed infections are believed to play a role (*5**,**20**,**21*). No interaction was seen in analysis by residential region, which is noteworthy given the time lag incurred by transporting samples to Riga for patients living elsewhere in Latvia, but a positive sign for the over-arching functionality of the TB program.

We made adjustments for temporal changes in our sensitivity analyses. It was reassuring to see a relatively steady time to treatment initiation among patients for whom Xpert was not used, particularly given the change in target populations. This study was restricted to MDR TB treatment cohorts, whereby inclusion in a particular year cohort is determined by the date on which treatment started. Use of Xpert could have also reduced the proportion of patients who did not start treatment because earlier use of treatment might have reduced the likelihood of death and pretreatment default.

Our study in Latvia suggests that implementing Xpert as an early-stage diagnostic tool from which treatment decisions are rapidly made reduces the time to MDR TB treatment initiation. This implementation not only probably benefits patients with MDR TB but might also reduce nosocomial and community transmission (*1*). Other countries should undertake similar research to evaluate the effect of Xpert on time to treatment initiation and treatment outcomes. Careful monitoring of time to treatment initiation could provide valuable performance data for national TB programs, around which targets could be set.

Although cost implications of introducing Xpert are variable in different settings, cost and affordability analyses suggest its viability as a diagnostic tool for detecting MDR TB, particularly in settings that have a high burden of MDR TB in which rifampin resistance has a good PPV for detecting MDR TB (*16**,**22*). Nevertheless, implementation of Xpert needs to be considered carefully in terms of how it is introduced into diagnostic algorithms, where machines are located, how clinicians interpret test results, and the common sites of TB in a particular population.
